# Supplementary material for: Identification of regulatory targets for the bacterial Nus factor complex
Source: Nat Commun. 2017 Dec 11;8:2027. doi: 10.1038/s41467-017-02124-9 (PMC5725501; doi:10.1038/s41467-017-02124-9)
Supplement: Supplementary file 1 — Supplementary Information [file 41467_2017_2124_MOESM1_ESM.pdf]

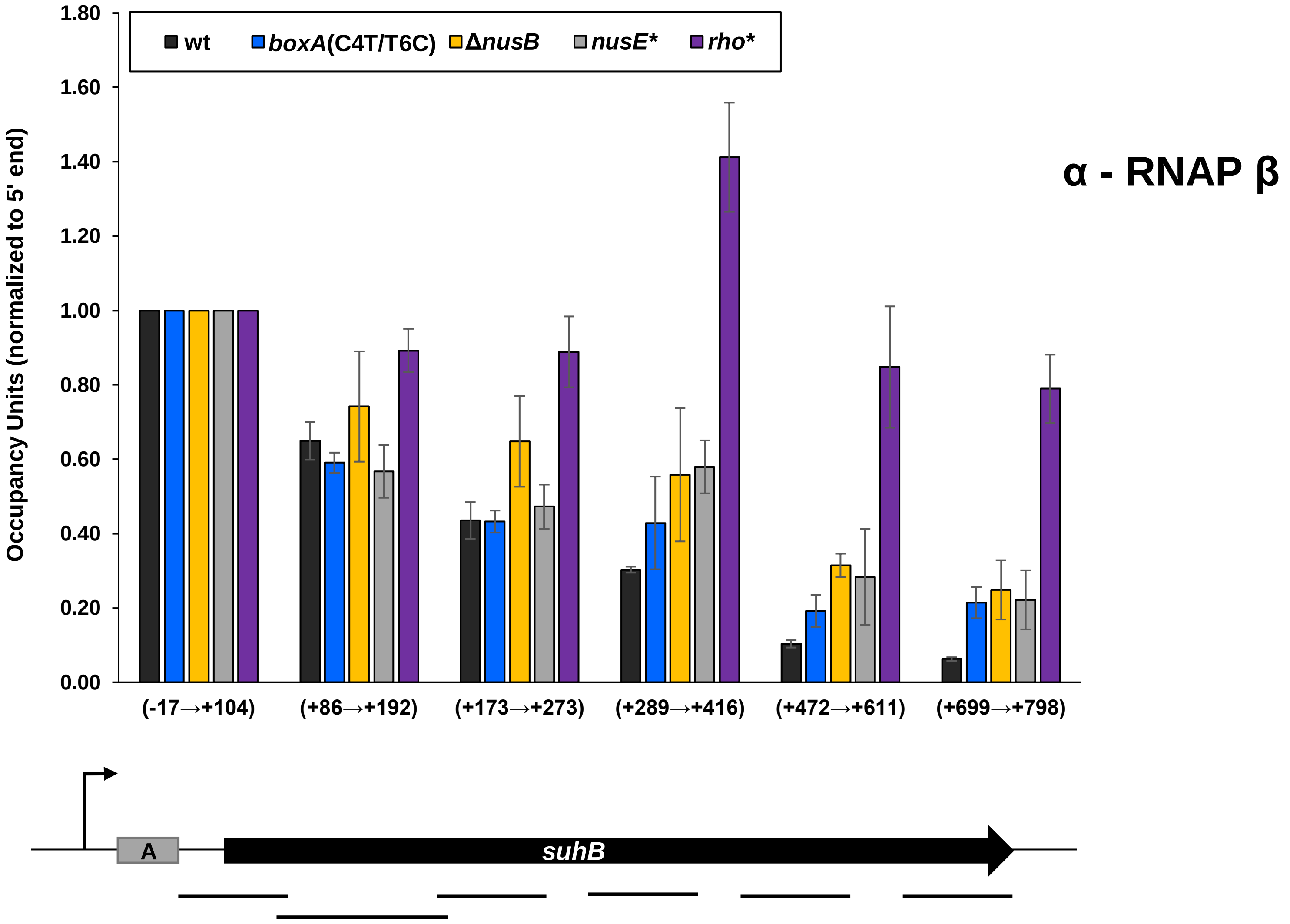

Supplementary Figure 1. Transcription termination within *suhB* is dependent on Rho and Nus factors. This is an extended version of Fig. 1. RNAP (β) enrichment at regions across *suhB* was measured using ChIP-qPCR in wt MG1655, *boxA*(C4T/T6C), Δ*nusB*, *nusE*(A12E) or *rho*(R66S) mutant strains. All values are normalised to the signal at the 5' end of *suhB* gene. *x*-axis labels indicate qPCR amplicon position relative to the *suhB* ORF. Error bars represent ±1 standard deviation from the mean (n=3). A schematic depicting *suhB* gene, the transcription start site (bent arrow) and *boxA* (grey rectangle) is shown below the graph. The six horizontal black lines indicate the position of the PCR amplicon.

```
Providencia_stuartii_MRSN_2154_uid162193                    -----ATCGCAAGAGTCTA----ACACAAAATTGCTTATTGTGACGAACAT---------
Erwinia_amylovora_ATCC_49946_uid46943                       --------CCTGCATCTTGCGGTGCAGAAAAGGGAAAAACCTTGCGCCGTA-------CC
Proteus_mirabilis_HI4320_uid61599                           -----ATCCATCTATTTTA----TCACAAATGTAGCCAAAAACGCGAACGC-------CT
Edwardsiella_ictaluri_93_146_uid59403                       ----TGCCAACGAGTATAA----AAACGGCAT--GGTTGTTTGCCGCACAC------TTT
Pantoea_ananatis_AJ13355_uid162073                          -ATGATGACGCGTACTCTA----ACATAAAGC-AGGACATTTACCGAACAC------CCT
Klebsiella_oxytoca_E718_uid170256                           ---TTATTCGTGCATGCTA----TCATAAAACGAAGACATAAGGAGATTTC------ACT
Enterobacter_638_uid58727                                   -------TCCGGCATATTA----TCATAAACGAGAGACATGATCCGAACTC------GCT
Cronobacter_sakazakii_ATCC_BAA_894_uid58145                 -----------GCATCTTA----TCATAAACCTGAGACATATTCCGAACCC------TCT
Citrobacter_koseri_ATCC_BAA_895_uid58143                    ----------GGCATATTA----TCATAAAACGGAGACATATTCCGAACTC------GCT
Escherichia_coli_K_12_substr__MG1655_uid57779               -GATTATTCACGCATCTTA----TCATAAAACGAAGACAGATGCCGATCTC------GCT
Shigella_flexneri_2a_2457T_uid57991                         -GATTATTCACGCATCTTA----TCATAAAACGAAGACAGATGCCGATCTC------GCT
Salmonella_enterica_serovar_Typhimurium_14028S_uid86059     -GATTATTCTGACATCTTA----CCATAAAACCAAGACAGATTCCGATCTC------GCT
Dickeya_dadantii_3937_uid52537                              ------CCGGCGTATGTTA----ACACAAACTTCTCTGTTTTCTCTTTCTCCCGCTATCT
Pectobacterium_atrosepticum_SCRI1043_uid57957               AGGAAACCGCCATACCTTA----ACACAAACTTAGCTATTTTACTTTTCTA-------CT
Sodalis_glossinidius__morsitans__uid58553                   ----GTGGGCAATATCGTA----GCATAGCCGTAGGTTTTTCGCTGACGCT-------CT
Photorhabdus_luminescens_laumondii_TTO1_uid61593            ------ATCAAATATCTTA----ACACAGCATTAGTCATTTTTCCGAATTA-------CT
Xenorhabdus_nematophila_ATCC_19061_uid49133                 ------GCCGAATATCTTA----ACACAGCATTAGTCATTTTGACGAAATA-------CT
Rahnella_aquatilis_CIP_78_65___ATCC_33071_uid86855          ---------CAACAGCCTA----ACACAACGGTAGTTAATTTTCAGAACCC-------CT
Serratia_AS12_uid67315                                      -GAAAATCACAGCATCTTA----ACACAGCCATAGGCATTTTGCCGAACAG-------CT
Yersinia_pestis_KIM_10_uid57875                             GGATAATCGCCATATCTTA----ACACAGACATAGGCATTTATCAGATCCC-------CT
                                                                                     *


Providencia_stuartii_MRSN_2154_uid162193                    -CTATA---TAATCCGCCCACTCAAAGTTT------TTCGTTCTTTAACATTCCAG-TGG
Erwinia_amylovora_ATCC_49946_uid46943                       GCTGCAAATATTCAGGATAA----------------CCCGTTCTTTAACATTC-AG-TGA
Proteus_mirabilis_HI4320_uid61599                           GTTATA---CTTCGCCTCGT--------TTTTTATCCCCGTTCTTTAACATCT-TG-TGG
Edwardsiella_ictaluri_93_146_uid59403                       GCTATA---CTCTGCGCCGTCTAAAT--TT------CCCGTTCTTTAACATCT-AG-TGG
Pantoea_ananatis_AJ13355_uid162073                          GCTATA---CTCCGCGCCGTTT------TC------CCCGTTCTTTAACATCC-AG-CGA
Klebsiella_oxytoca_E718_uid170256                           GTTACT---ATATGCGCCGTTTT-----TT------CCCGTTCTTTAACATCC-AG-TGA
Enterobacter_638_uid58727                                   GCTATA---CTCTGCGCCGTT-------TTCGTTTCCCTGTTCTTTAACATCC-AG-TGA
Cronobacter_sakazakii_ATCC_BAA_894_uid58145                 GCTATA---ATCCGCGCCGTT-------TT------CCCGTTCTTTAACATCC-AG-TGA
Citrobacter_koseri_ATCC_BAA_895_uid58143                    GCTATA---CTCTGCGCCGTT-------TT------CCCGTTCTTTAACATCC-AG-TGA
Escherichia_coli_K_12_substr__MG1655_uid57779               GCTATA---CTCTGCGCCGTT-------TT------CCCGTTCTTTAACATCC-AG-TGA
Shigella_flexneri_2a_2457T_uid57991                         GCTATA---CTCTGCGCCGTT-------TT------CCCGTTCTTTAACATCC-AG-TGA
Salmonella_enterica_serovar_Typhimurium_14028S_uid86059     GCTATA---CTCTGCGCCGTT-------TT------CCCGTTCTTTAACATCC-AG-TGA
Dickeya_dadantii_3937_uid52537                              GCTATA---CTCCGCGCCGT--------TT------TCTGTTCTTTAACATTCTTG-TGG
Pectobacterium_atrosepticum_SCRI1043_uid57957               GCTATA---CTCTGCGCCGC--------TT------TCCGTTCTTTAACATTCCAG-TGG
Sodalis_glossinidius__morsitans__uid58553                   GCTATACTCCTCCGCGCTG---------TT------TTAGTTCTTTAACATTCTAG-TGG
Photorhabdus_luminescens_laumondii_TTO1_uid61593            GCTATA---CTACGCGCCGATTAAT---TA------CCCGTTCTTTAACATCCTGG-TGG
Xenorhabdus_nematophila_ATCC_19061_uid49133                 GCTATA---CTGCGCGCCGATTTGT---TT------CCCGTTCTTTAAAATTCTGG-TGG
Rahnella_aquatilis_CIP_78_65___ATCC_33071_uid86855          GCTATA---CTGCGCGCCGTTTCCCGTATT------CCCGTTCTTTAACATCCTAGTTGG
Serratia_AS12_uid67315                                      GCTATA---CTTCGCGCCGT--------TT------CCTGTTCTTTAACATCCTAGTTGG
Yersinia_pestis_KIM_10_uid57875                             GCTATA---CTGTGCGCCGT--------TT------CTCGTTCTTTAACATCCTAG-TGG
                                                              *                                    ********* **    *  *
```

```
Providencia_stuartii_MRSN_2154_uid162193                    --A-AAAAGCCC----------
Erwinia_amylovora_ATCC_49946_uid46943                       --GACGATATTACCT-------
Proteus_mirabilis_HI4320_uid61599                           --A-AGATAACC----------
Edwardsiella_ictaluri_93_146_uid59403                       --A-AGATTCCC----------
Pantoea_ananatis_AJ13355_uid162073                          --G-AGATACCG----------
Klebsiella_oxytoca_E718_uid170256                           --G-AGATACCG----------
Enterobacter_638_uid58727                                   --G-AGTAACCG----------
Cronobacter_sakazakii_ATCC_BAA_894_uid58145                 --G-AGAAACCTGATGCATCCG
Citrobacter_koseri_ATCC_BAA_895_uid58143                    --G-AGAGATCG-ATGCATCCG
Escherichia_coli_K_12_substr__MG1655_uid57779               --G-AGAGACCG----------
Shigella_flexneri_2a_2457T_uid57991                         --G-AGAGACCG----------
Salmonella_enterica_serovar_Typhimurium_14028S_uid86059     --G-AGAGACCG----------
Dickeya_dadantii_3937_uid52537                              --A-CGATACC-----------
Pectobacterium_atrosepticum_SCRI1043_uid57957               --ACCGATACC-----------
Sodalis_glossinidius__morsitans__uid58553                   ACA-CGATATCC----------
Photorhabdus_luminescens_laumondii_TTO1_uid61593            --A-AGATATCCC---------
Xenorhabdus_nematophila_ATCC_19061_uid49133                 --A-AGATACCCC---------
Rahnella_aquatilis_CIP_78_65___ATCC_33071_uid86855          --A-AGATATCC----------
Serratia_AS12_uid67315                                      --A-AGATACCC----------
Yersinia_pestis_KIM_10_uid57875                             --A-AGATACCC----------
```

Supplementary Figure 2. MUSCLE (v3.8) Alignment (CLUSTAL Format) of 100 bp regions upstream of *suhB* homologues in *Enterobacteriaceae* species. Species names are indicated to the left of the alignment. Asterisks indicate positions that are 100% conserved across the 20 species.

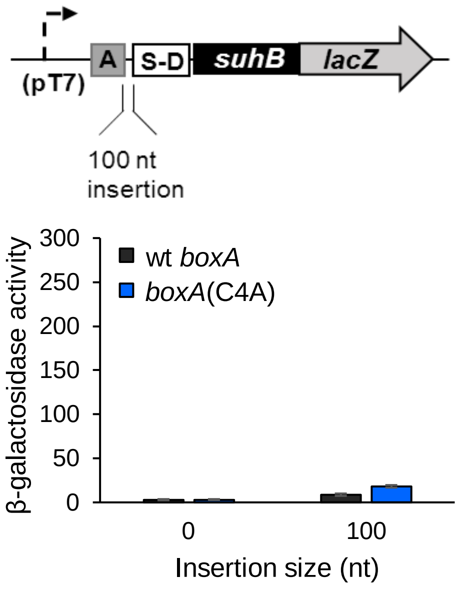

Supplementary Figure 3. The effect of 100 nt insertion between *boxA* and the S-D on *suhB-lacZ* expression levels when the native promoter is absent. β–galactosidase assay of wild-type ("wt *boxA*"; dark grey bars) and *boxA* mutant (*boxA*(C4A); blue bars) *suhB* translational fusion to *lacZ*. The length of inserted sequence is indicated on the *x*-axis. The native *suhB* promoter was replaced by a T7 promoter ("pT7"; see schematic above the graph). Additionally, bacterial cells carried an empty pBAD18 vector (T7 RNAP was not supplied in this assay). Cells were grown in the presence of 0.2% arabinose. Error bars represent ±1 standard deviation from the mean (n=3). Note that the β-galactosidase activity from the translational fusion construct shown here is substantially lower than the activity from the equivalent transcriptional fusion construct with a native *suhB* promoter (Fig. 5, far right). Moreover, β-galactosidase activity from a wild-type *suhB-lacZ* translational fusion construct is ~8-fold higher than the activity from the equivalent transcriptional fusion construct (607±8 and 78±3 β-galactosidase activity units, respectively). We conclude that the majority of β-galactosidase activity for the *suhB-lacZ* transcriptional fusion with a native promoter and a 100 nt insertion (Fig. 5, far right) is due to transcription from the native promoter.

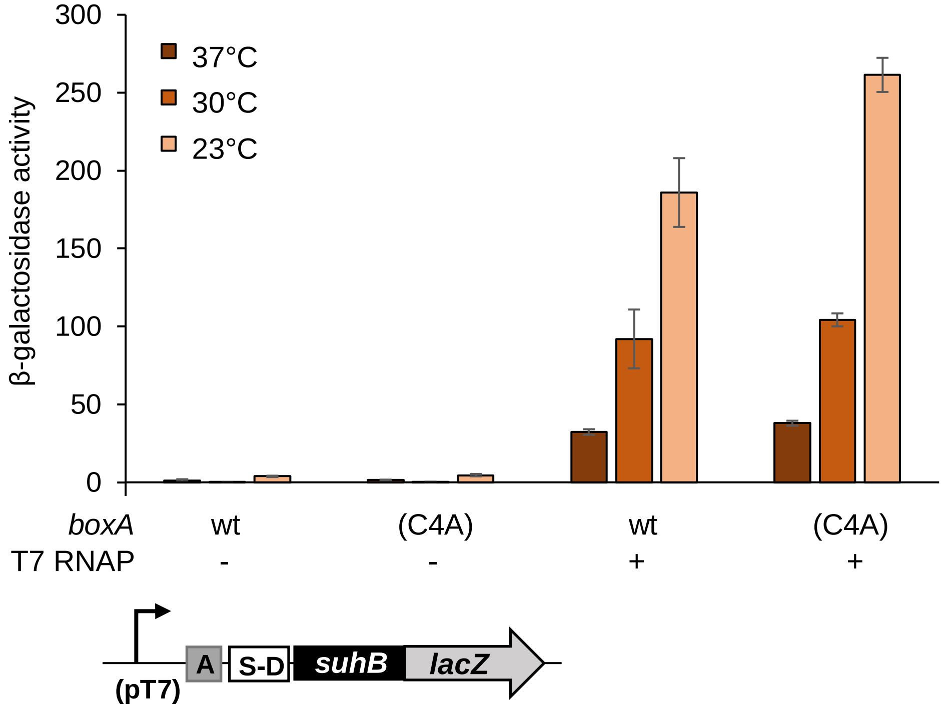

Supplementary Figure 4. *suhB* expression by T7 RNAP abolishes BoxA-mediated translational repression. β–galactosidase assay of wild-type ("wt") and *boxA* mutant ("C4A") *suhB* translational fusions to *lacZ*. The native *suhB* promoter was replaced by a T7 promoter ("pT7"; see schematic below the graph). Additionally, bacterial cells carried a plasmid with either an empty pBAD18 vector or pBAD18 expressing T7 RNAP (as indicated on the *x*-axis). β-galactosidase activity was measured for cells grown in the presence of 0.2% arabinose to induce T7 RNAP expression at 37 °C, 30 °C or 23 °C as indicated in the legend. Error bars represent ±1 standard deviation from the mean (n=3).

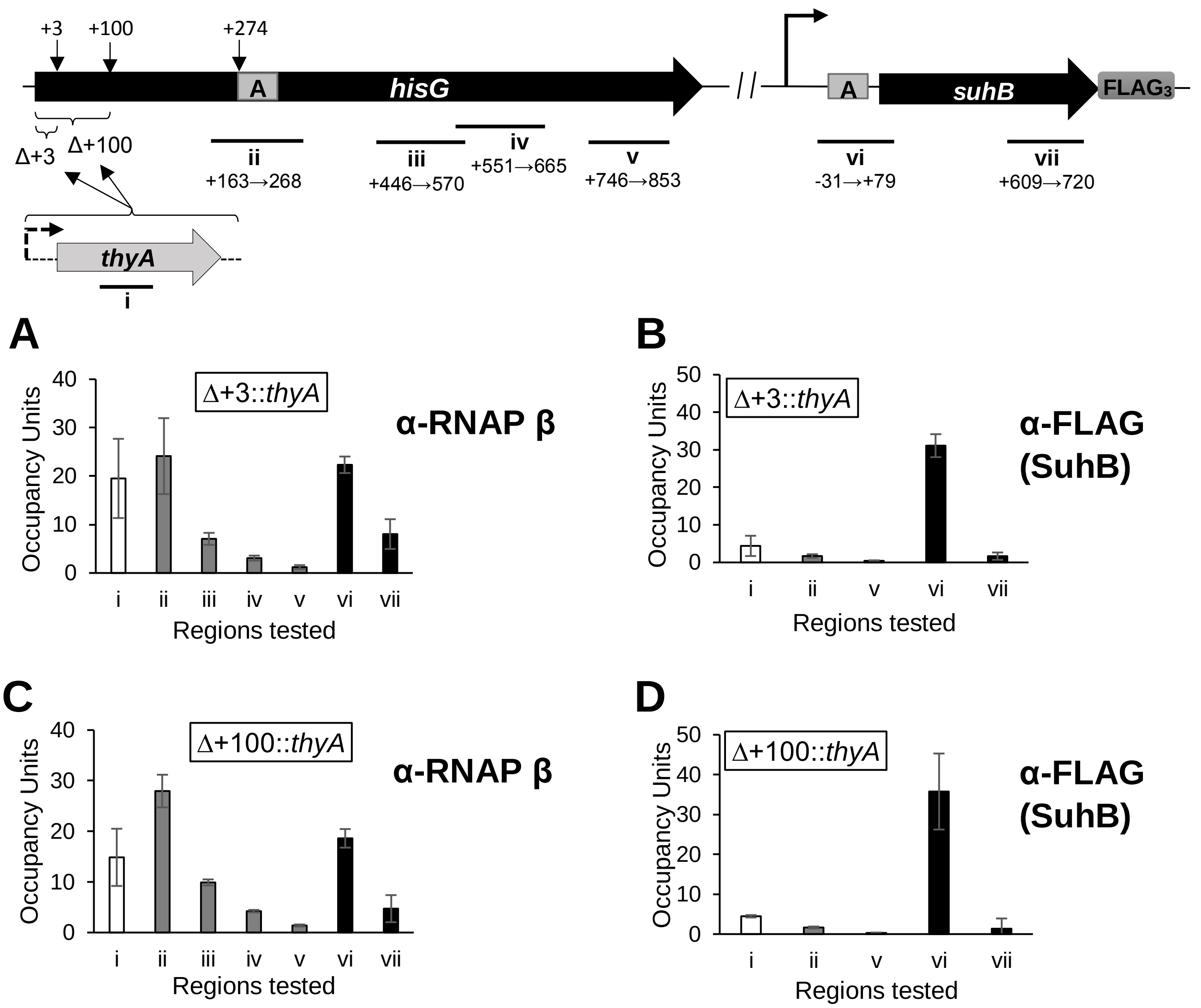

Supplementary Figure 5. Evidence for and against functional BoxA elements upstream of *suhB* and inside *hisG* gene in *S.* Typhimurium, respectively. Previous studies reported a functional BoxA within the *S.* Typhimurium *hisG* mRNA [1,2]. This putative BoxA was reported as being functional only when *hisG* translation was abolished by mutation of the gene. Hence, we interrupted *hisG* upstream of the putative *boxA* by inserting the *thyA* gene 3 bp or 100 bp downstream of the start codon. RNAP (β) **(A and C)** and SuhB-FLAG **(B and D)** association with *thyA*, *hisG* and *suhB* was measured using ChIP-qPCR in a derivative of *S.* Typhimurium strain 14028s in which *thyA* was deleted at its native locus, and *hisG* was disrupted by insertion of *thyA*, replacing the first 3 **(A and B)** or 100 **(C and D)** nucleotides of *hisG*. *x*-axis labels indicate the qPCR amplicon used, with numbers corresponding to the schematics above the graphs. Error bars represent ±1 standard deviation from the mean (n=3). In the schematic, the *suhB boxA* and the putative *hisG boxA* are indicated by grey rectangles. Numbers above the arrows represent nucleotide positions relative to the *hisG* gene start (without *thyA* insertion). Horizontal black lines indicate the positions of PCR amplicons.

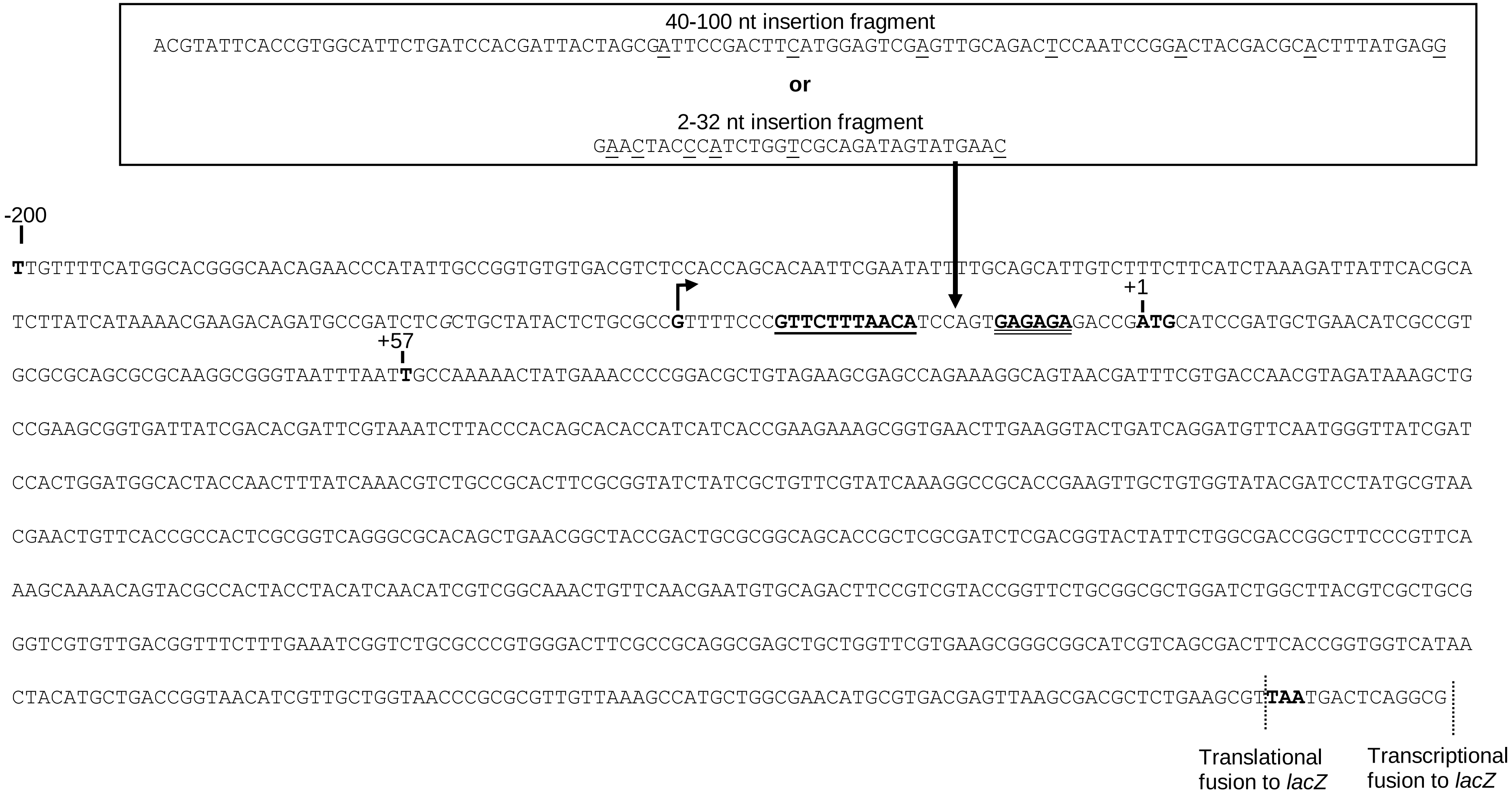

Supplementary Figure 6. *suhB* gene sequence used in *lacZ* fusion constructs. Relevant features used in this work are indicated: transcription start site (bent arrow), *boxA* sequence (single underline), S-D (double underline), start ("ATG") and stop ("TAA") codons (bold). The non-coding DNA sequence inserted between BoxA and S-D (Fig. 5) is shown in the box above, and the arrow points to the position of the insertion. Underlined nucleotides indicate the 3' ends of various insertions and correspond to the insertion size labelled in Fig. 5. *suhB* sequence used in *lacZ* fusions in Fig. 3A-B included from position -200 to the end of the gene, as indicated by dashed lines. The short *suhB*-*lacZ* transcriptional fusion from Fig. 4B included *suhB* sequence up to position +57, and an in-frame stop codon immediately after the gene fragment.

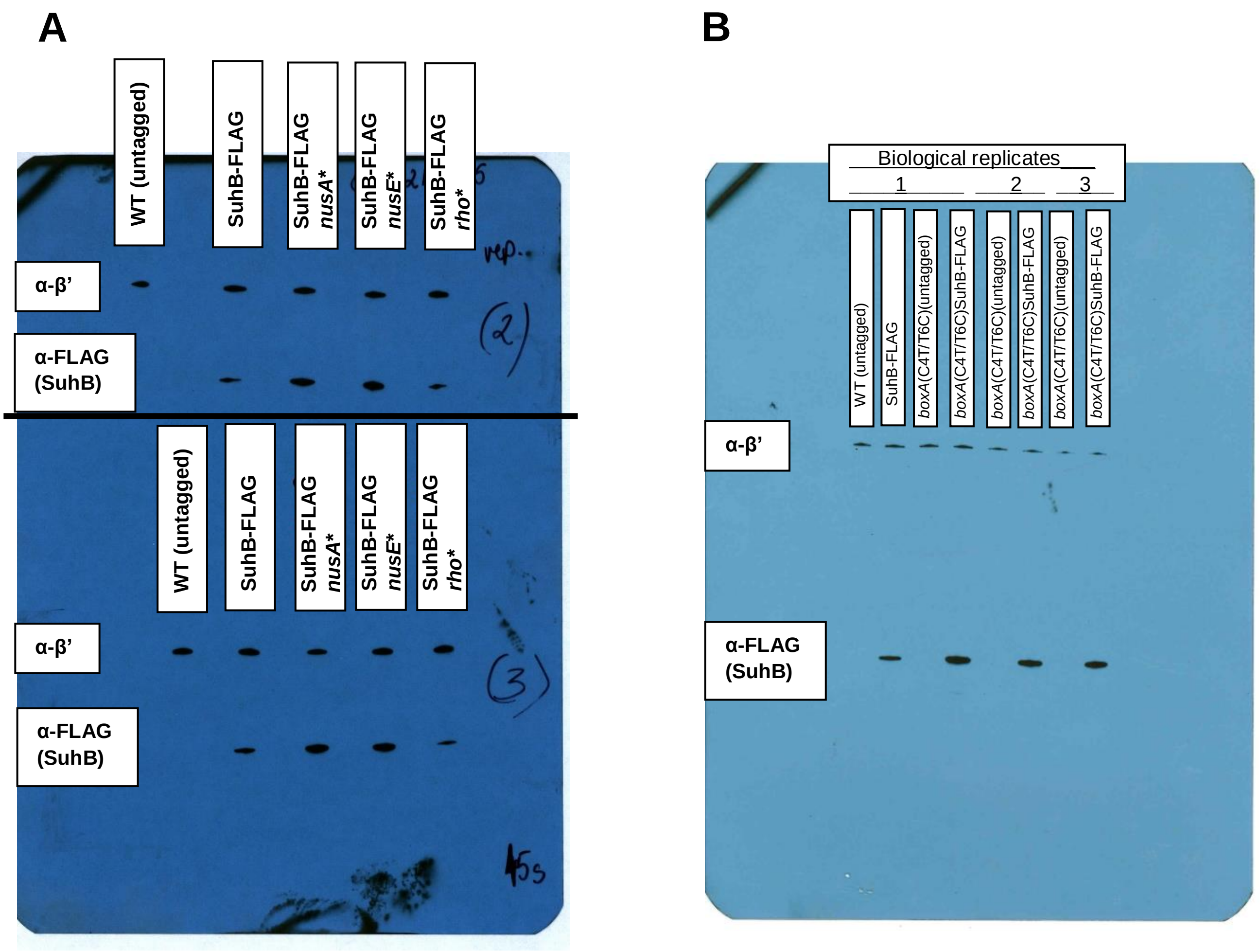

Supplementary Figure 7. Unprocessed western blot images from Fig. 3D-C. Western blots showing SuhB-FLAG protein levels in wild-type cells, *nusAcs10*, *nusE*(A12E), *rho*(R66S) **(A)**, and *boxA*(C4T/T6C) mutants **(B)**. SuhB-FLAG was probed with α-FLAG antibody; RNAP β' was probed as a loading control. Blots from the same SDS-PAGE gel were processed in parallel, see 'Methods' for details.

Supplementary Table 1. List of *nusB, nusE* and *nusG* mutants isolated in the genetic selection for factors that repress *suhB*.

	Nucleotide Change[a]	Amino Acid Change[a]	Other
nusB	C22A	R8S	N/A
	C26A	A9D	N/A
	T52A	Y18N	N/A
	T52G	Y18D	N/A
	A53C	Y18S	N/A
	A53G	Y18C	N/A
	C320A	A107E	N/A
	A322T	I108F	N/A
	G344A	G115D	N/A
	T377A	V126E	N/A
	T9G	Silent	N/A
	T18G	Silent	N/A
	C21G	Silent	N/A
	N/A	N/A	IS3 insertion after position 140
nusE	A7C	N3H	N/A
	C35A*	A12E*	N/A
	T53A*	I18N*	N/A
	A56T	D19V	N/A
	C295A	Q99K	N/A
	G297T*	Q99H*	N/A
	A15G	Silent	N/A
	G294A	Silent	N/A
	C300A	Silent	N/A
	C-79T	N/A	N/A
	A-12G	N/A	N/A
nusG	N/A	N/A	Duplication of -23 to -2
	ΔA515	N/A	N/A
	N/A	N/A	IS1 insertion after position 534

[a] mutations marked with an asterisk were isolated independently on two occasions

Supplementary Table 2. A list of relevant *boxA* sequences from *E. coli* and related bacteria.

**Species**	**Gene**	***boxA* sequence and nt positions[a]**										
		**1**	**2**	**3**	**4**	**5**	**6**	**7**	**8**	**9**	**10**	**11**
E. coli, S. enterica and C. koseri	rRNA	G	C	U	C	U	U	U	A	A	C	A
	suhB	G	U	U	C	U	U	U	A	A	C	A
	suhB(C4A)	G	U	U	**A**	U	U	U	A	A	C	A
	suhB(C4T/T6C)	G	U	U	**U**	U	**C**	U	A	A	C	A
C. koseri	*CKO_00699*	G	C	U	C	U	U	U	A	A	C	A
	CKO_00699(C4A)	G	C	U	**A**	U	U	U	A	A	C	A
S. enterica	*hisG*	G	C	U	A	U	U	U	A	A	C	C

[a] Nucleotide positions are numbered 1-11 above the sequences. BoxA from rRNA is considered a consensus. A critical nucleotide important for Nus factor association is “C” at position 4 [3,4], and the mismatch in the *S. enterica* putative *hisG* BoxA sequence is underlined. *suhB* and *CKO_00699 boxA* mutations used in this study are in bold.

Supplementary Table 3. List of bacterial strains and plasmids used in this study.

Name	Description	Source
	**Strains**	
MG1655	MG1655 (F- λ- Δ*ilvG rfb*-50 *rph*-1)	[5]
EPI300	*F⁻ mcrA Δ(mrr-hsdRMS-mcrBC) Φ80dlacZΔM15 ΔlacX74 recA1 endA1 araD139 Δ(ara, leu)7697 galU galK λ- rpsL (StrR) nupG trfA dhfr*	Epicentre
AMD054	MG1655Δ*lacZ*	[6]
JW023	MG1655Δ*lacZ*Δ*nusB::thyA*	This study
GB003	MG1655Δ*lacZ nusE(A12E)*	This study
GB004	MG1655Δ*lacZ rho(R66S)*	This study
VS066	MG1655 *suhB*-FLAG	[7]
GB006	MG1655 *nusE(A12E) suhB*-FLAG	This study
GB007	MG1655 *rho(R66S) suhB*-FLAG	This study
GB023	MG1655 *suhB*(*boxA*(C4T/T6C))	This study
GB024	MG1655 *suhB*(*boxA*(C4T/T6C))-FLAG	This study
GB036	14028S Δ*+3(hisG)::thyA suhB*-FLAG	This study
GB037	14028S Δ*+100(hisG)::thyA suhB*-FLAG	This study
	**Plasmids**	
pAMD-BA-lacZ	Single-copy *lacZ* expression vector	[6]
pKD46	Encodes λ recombinase system	[8]
pJTW067	*suhB*(T607C) +200nt upstream transcriptional fusion to *lacZ*	This study
pGB1	*suhB* +200nt upstream transcriptional fusion to *lacZ*	This study
pGB2	*suhB*(*boxA*(C4A)) +200nt upstream transcriptional fusion to *lacZ*	This study
pGB67	*suhB*(*boxA*(C4T/T6C)) +200nt upstream transcriptional fusion to *lacZ*	This study
pGB3	*suhB* +200nt upstream translational fusion to *lacZ*	This study
pGB4	*suhB*(*boxA*(C4A)) +200nt upstream translational fusion to *lacZ*	This study
pGB68	*suhB*(*boxA*(C4T/T6C)) +200nt upstream translational fusion to *lacZ*	This study
pGB7	*boxA*-2nt-SD *suhB* +200nt upstream transcriptional fusion to *lacZ*	This study
pGB8	*boxA*-4nt-SD *suhB* +200nt upstream transcriptional fusion to *lacZ*	This study
pGB9	*boxA*-8nt-SD *suhB* +200nt upstream transcriptional fusion to *lacZ*	This study
pGB10	*boxA*-10nt-SD *suhB* +200nt upstream transcriptional fusion to *lacZ*	This study
pGB11	*boxA*-16nt-SD *suhB* +200nt upstream transcriptional fusion to *lacZ*	This study

pGB12	*boxA*-32nt-SD *suhB* +200nt upstream transcriptional fusion to *lacZ*	This study
pGB13	*boxA*-40nt-SD *suhB* +200nt upstream transcriptional fusion to *lacZ*	This study
pGB14	*boxA*-50nt-SD *suhB* +200nt upstream transcriptional fusion to *lacZ*	This study
pGB15	*boxA*-60nt-SD *suhB* +200nt upstream transcriptional fusion to *lacZ*	This study
pGB16	*boxA*-70nt-SD *suhB* +200nt upstream transcriptional fusion to *lacZ*	This study
pGB17	*boxA*-80nt-SD *suhB* +200nt upstream transcriptional fusion to *lacZ*	This study
pGB18	*boxA*-90nt-SD *suhB* +200nt upstream transcriptional fusion to *lacZ*	This study
pGB19	*boxA*-100nt-SD *suhB* +200nt upstream transcriptional fusion to *lacZ*	This study
pGB23	*boxA*(C4A)-2nt-SD *suhB* +200nt upstream transcriptional fusion to *lacZ*	This study
pGB24	*boxA*(C4A)-4nt-SD *suhB* +200nt upstream transcriptional fusion to *lacZ*	This study
pGB25	*boxA*(C4A)-8nt-SD *suhB* +200nt upstream transcriptional fusion to *lacZ*	This study
pGB26	*boxA*(C4A)-10nt-SD *suhB* transcriptional fusion to *lacZ*	This study
pGB27	*boxA*(C4A)-16nt-SD *suhB* transcriptional fusion to *lacZ*	This study
pGB29	*boxA*(C4A)-32nt-SD *suhB* transcriptional fusion to *lacZ*	This study
pGB30	*boxA*(C4A)-40nt-SD *suhB* +200nt upstream transcriptional fusion to *lacZ*	This study
pGB31	*boxA*(C4A)-50nt-SD *suhB* +200nt upstream transcriptional fusion to *lacZ*	This study
pGB32	*boxA*(C4A)-60nt-SD *suhB* +200nt upstream transcriptional fusion to *lacZ*	This study
pGB33	*boxA*(C4A)-70nt-SD *suhB* +200nt upstream transcriptional fusion to *lacZ*	This study
pGB34	*boxA*(C4A)-80nt-SD *suhB* +200nt upstream transcriptional fusion to *lacZ*	This study
pGB35	*boxA*(C4A)-90nt-SD *suhB* +200nt upstream transcriptional fusion to *lacZ*	This study
pGB36	*boxA*(C4A)-100nt-SD *suhB* +200nt upstream transcriptional fusion to *lacZ*	This study
pBAD18	Contains arabinose inducible promoter	[9]
pGB115	pBAD18:T7RNAP	This study
pGB84	pT7-*suhB* translational fusion to *lacZ*	This study
pGB83	pT7-*boxA*(C4A)-*suhB* translational fusion to *lacZ*	This study
pGB94	pT7-*boxA*-100nt-*suhB* translational fusion to *lacZ*	This study
pGB95	pT7-*boxA*(C4A)-100nt-*suhB* translational fusion to *lacZ*	This study
pGB109	pHigh-*CKO_00699*(R82A) transcriptional fusion to *lacZ*	This study
pGB110	pHigh-*CKO_00699*(R82A)-*boxA*(C4A) transcriptional fusion to *lacZ*	This study
pGB186	*suhB* (A1C, T2A) +200nt upstream transcriptional fusion to *lacZ*	This study
pGB187	*suhB*(*boxA*(C4T/T6C)) (A1C, T2A) +200nt upstream transcriptional fusion to *lacZ*	This study
pGB193	*suhB* (A1C, T2A) (-200→+57nt) transcriptional fusion to *lacZ*	This study
pGB192	*suhB*(*boxA*(C4T/T6C)) (A1C, T2A) (-200→+57nt) transcriptional fusion to *lacZ*	This study

Supplementary Table 4. List of oligonucleotides used in this study.

Name	Sequence	Description
JW125	AAGCGAAAATCGGCAATA	qPCR: *bglB* fwd
JW126	CATGGCCTGCAACATATC	qPCR: *bglB* rev
JW595	GGGCCAGTGTATGGTAAA	qPCR: *thyA* mid fwd
JW663	TATACTCTGCGCCGTTTT	qPCR: *suhB* 5' end fwd
JW664	CCGGGGTTTCATAGTTTT	qPCR: *suhB* 5' end rev
JW665	CCGTTCAAAGCAAAACAG	qPCR: *suhB* (+472→611) fwd
JW666	AAGAAACCGTCAACACGA	qPCR: *suhB* (+472→611) rev
JW1495	ACCCGTTCGATGTTGTTA	qPCR: *sbcC* (14028S) fwd
JW1496	TCTGCCCGTAAATCTCAG	qPCR: *sbcC* (14028S) rev
JW3415	GGACGGATCCTCGAGCATGCTAGACAGCTGCATGCATCTTTG	Cloning: pHigh fwd into pAMD-BA-lacZ fwd
JW3605	GGACGGATCCTCGAGCATGCTTGTTTTCATGGCACGGG	Cloning: *suhB* + 200nt ups. fwd
JW3607	TTCATGCATTGCTAGCCGCCTGAGTCATTAACGC	Cloning: *suhB* transciptional fusion to *lacZ* rev
JW6036	GGCCAGTGCCAAGCTTGCACGCTTCAGAGCGTCGC	Cloning: *suhB* translational fusion to *lacZ* rev
JW6038	TCACTGGATGTTAAATAACGGGAAAACGGC	Cloning: *boxA*(C4A) mutation rev
JW6039	TTTAACATCCAGTGAGAGAGACCG	Cloning: *boxA*(C4A) mutation fwd
JW6505	CAGATGCCGATCTCGCTGCTATACTCTGCGCCGTTTTCCCGTTTAGACAGCTGCATGCAT	FRUIT: TU *suhB*/*boxA E. coli*
JW6506	TGTTCAGCATCGGATGCATCGGTCTCTCTCACTGGATGTTAAAGGTGTAGGCTGGAGCTG	FRUIT: TD *suhB*/*boxA E. coli*
JW6596	AACATCCGAAGTGAGAGAGACCGATG	Cloning: 2nt insert between *boxA* and S-D fwd
JW6597	TCTCTCTCACTTCGGATGTTAAAGAACGGG	Cloning: 2nt insert between *boxA* and S-D rev
JW6598	TAACATCCGAACAGTGAGAGAGACCGATGC	Cloning: 4nt insert between *boxA* and S-D fwd
JW6599	TCTCTCTCACTGTTCGGATGTTAAAGAACGGG	Cloning: 4nt insert between *boxA* and S-D rev
JW6600	CTTTAACATCCGAACTACCAGTGAGAGAGACCGATGC	Cloning: 8nt insert between *boxA* and S-D fwd
JW6601	TCTCTCACTGGTAGTTCGGATGTTAAAGAACGGG	Cloning: 8nt insert between *boxA* and S-D rev
JW6602	GAACTACCCATCTGGTAGTGAGAGAGACCGATGCATC	Cloning: 16nt insert between *boxA* and S-D fwd
JW6603	ACCAGATGGGTAGTTCGGATGTTAAAGAACGGGAAAAC	Cloning: 16nt insert between *boxA* and S-D rev
JW6604	GAACTACCCATCTGGTCGCAGATAGTATGAACAGTGAGAGAGACCGATGCATC	Cloning: 32nt insert between *boxA* and S-D fwd
JW6605	GTTCATACTATCTGCGACCAGATGGGTAGTTCGGATGTTAAAGAACGGGAAAAC	Cloning: 32nt insert between *boxA* and S-D rev
JW7070	CGTTCTTTAACATCCACGTATTCACCGTGGCATTC	Cloning: 100nt insert after *boxA* fwd

JW7071	GAATGCCACGGTGAATACGTGGATGTTAAAGAACGGGAAAACG	Cloning: 100nt insert after *boxA* rev
JW7072	TCGGTCTCTCTCACTCCTCATAAAGTGCGTCGTAG	Cloning: 100nt insert before S-D rev
JW7073	ACGCACTTTATGAGGAGTGAGAGAGACCGATGCATC	Cloning: 100nt insert before S-D fwd
JW7184	TCTCTCTCACTTCGGATGTTAAATAACGGG	Cloning: 2nt insert between *boxA*(C4A) and S-D rev
JW7185	TCTCTCTCACTGTTCGGATGTTAAATAACGGG	Cloning: 4nt insert between *boxA*(C4A) and S-D rev
JW7186	ATTTAACATCCGAACTACCAGTGAGAGAGACCGATGC	Cloning: 8nt insert between *boxA*(C4A) and S-D fwd
JW7187	TCTCTCACTGGTAGTTCGGATGTTAAATAACGGG	Cloning: 8nt insert between *boxA*(C4A) and S-D rev
JW7188	CATCCGAACTACCCAAGTGAGAGAGACCGATGC	Cloning: 10nt insert between *boxA* and S-D fwd
JW7189	TCACTTGGGTAGTTCGGATGTTAAAGAACGGGAAAAC	Cloning: 10nt insert between *boxA* and S-D rev
JW7190	TCACTTGGGTAGTTCGGATGTTAAATAACGGGAAAAC	Cloning: 10nt insert between *boxA*(C4A) and S-D rev
JW7191	ACCAGATGGGTAGTTCGGATGTTAAATAACGGGAAAAC	Cloning: 16nt insert between *boxA*(C4A) and S-D rev
JW7195	GTTCATACTATCTGCGACCAGATGGGTAGTTCGGATGTTAAATAACGGGAAAAC	Cloning: 32nt insert between *boxA*(C4A) and S-D rev
JW7196	TGGCATTCTGATCCACGATTACTAGCGAAGTGAGAGAGACCGATGCATC	Cloning: 40nt insert between *boxA* and S-D fwd
JW7197	TGGATCAGAATGCCACGGTGAATACGTGGATGTTAAAGAACGGGAAAAC	Cloning: 40nt insert between *boxA* and SD Rev
JW7198	TGGATCAGAATGCCACGGTGAATACGTGGATGTTAAATAACGGGAAAAC	Cloning: 40nt insert between *boxA*(C4A) and SD rev
JW7199	CGTTATTTAACATCCACGTATTCACCGTGGCATTC	Cloning: 50-100nt insert after *boxA*(C4A) Fwd
JW7200	GAATGCCACGGTGAATACGTGGATGTTAAATAACGGGAAAACG	Cloning: 50-100nt insert after *boxA*(C4A) Rev
JW7201	AGCGATTCCGACTTCAGTGAGAGAGACCGATGCATC	Cloning: 50nt insert between *boxA* and S-D fwd
JW7202	TCGGTCTCTCTCACTGAAGTCGGAATCGCTAGTAATC	Cloning: 50nt insert between *boxA* and S-D rev
JW7203	ACTTCATGGAGTCGAAGTGAGAGAGACCGATGCATC	Cloning: 60nt insert between *boxA* and S-D fwd
JW7204	TCGGTCTCTCTCACTTCGACTCCATGAAGTCGGAATC	Cloning: 60nt insert between *boxA* and S-D rev
JW7205	GTCGAGTTGCAGACTAGTGAGAGAGACCGATGCATC	Cloning: 70nt insert between *boxA* and S-D fwd
JW7206	TCGGTCTCTCTCACTAGTCTGCAACTCGACTCCATG	Cloning: 70nt insert between *boxA* and S-D rev
JW7207	AGACTCCAATCCGGAAGTGAGAGAGACCGATGCATC	Cloning: 80nt insert between *boxA* and S-D fwd
JW7208	TCGGTCTCTCTCACTTCCGGATTGGAGTCTGCAAC	Cloning: 80nt insert between *boxA* and S-D rev
JW7209	CCGGACTACGACGCAAGTGAGAGAGACCGATGCATC	Cloning: 90nt insert between *boxA* and S-D fwd
JW7210	TCGGTCTCTCTCACTTGCGTCGTAGTCCGGATTG	Cloning: 90nt insert between *boxA* and S-D rev
JW7674	GCCGTTTTCCCGTTTTCTAACATCCAGTGAGA	Cloning: MU *suhB*/*boxA*(C4T/T6C)
JW7675	TCTCACTGGATGTTAGAAAACGGGAAAACGGC	Cloning: MD *suhB*/*boxA*(C4T/T6C)
JW7793	GGCGTTACTGGTGTTATTTC	qPCR: *ynbB* fwd
JW7794	CAGACCTTCCAATGTTTTTC	qPCR: *ynbB* rev
JW7848	TCATAACTACATGCTGACCG	qPCR: *suhB* 3' (+699→+798) fwd
JW7849	CTTCAGAGCGTCGCTTAAC	qPCR: *suhB* 3' (+699→+798) rev
JW7907	CTTACCCACAGCACACCATC	qPCR: *suhB*(+172→+273) fwd

JW7908	GTTGGTAGTGCCATCCAGTG	qPCR: *suhB*(+172→+273) rev
JW7926	CCGCACTTCGCGGTATCTAT	qPCR: *suhB*(+289→+416) fwd
JW7927	CGGTAGCCGTTCAGCTGTG	qPCR: *suhB*(+289→+416) rev
JW7934	TAGAAGCGAGCCAGAAAGGC	qPCR: *suhB*(+85→+192) fwd
JW7935	GATGGTGTGCTGTGGGTAAGA	qPCR: *suhB*(+85→+192) rev
JW8021	GGACGGATCCTCGAGCATGCTAATACGACTCACTATAGTTTTCCCGTTCTTTAACATC	Cloning: pT7-*suhB* fwd
JW8022	GGACGGATCCTCGAGCATGCTAATACGACTCACTATAGTTTTCCCGTTATTTAACATC	Cloning: pT7-*boxA*(C4A)-*suhB* fwd
JW8284	AGGATCCCCGGGTACCTTACGCGAACGCGAAGTC	Cloning: T7RNAP into pBAD18 rev
JW8320	TTCATGCATTGCTAGC	Cloning: *CKO_00699*(R82A) transcript. *lacZ* fusion rev
JW8325	CAAATTGTTAAAtAGCACACCCTAC	Cloning: *CKO_00699*(R82A) *boxA*(C4A) rev
JW8326	GCTaTTTAACAATTTGTTTTACAGGGGATG	Cloning: *CKO_00699*(R82A) *boxA*(C4A) fwd
JW8340	GTTTTTTTGGGCTAGCGAAGGAGATATACATATGAACACGATTAACATCGCTAAG	Cloning: T7RNAP into pBAD18 fwd
JW8355	GCGCGATACAGACCGGTTCAGACAGGATAAAGAGGAACGCAGATAGACAGCTGCATGCAT	FRUIT: *thyA* → +1 of *hisG* 14028S fwd
JW8356	CGGCCTGATTTCTGAATAGCTATGCGTAAGCGGGTGTTGTCTAAGTGTAGGCTGGAGCTG	FRUIT: *thyA* → +3 of *hisG* 14028S rev
JW8357	AATCGGCATGTTTTCCGCCATCGCAATCAGGCGCTGAGTGTGTAGTGTAGGCTGGAGCTG	FRUIT: *thyA* → +100 of *hisG* 14028S rev
JW8371	CCCGTTCTTTAACATCCAGT	qPCR: *suhB*(-31→+79) fwd
JW8372	CCGGAGTTTCATAGTTTTTGG	qPCR: *suhB*(-31→+79) rev
JW8375	CTTTGAAATTGGCCTTCGTC	qPCR: *suhB*(+609→720) fwd
JW8376	ACCGGTCATCATGTAGTTATG	qPCR: *suhB*(+609→720) rev
JW8377	GATGATGACATTCCGGGTC	qPCR: *hisG*(+163→+286) fwd
JW8378	GGGTTAAATAGCGTGGATCT	qPCR: *hisG*(+163→+286) rev
JW8385	AATCCGGGTCGTTTTTCAG	qPCR: *thyA* mid rev
JW8383	AAAGGCCGACAATTCTGC	qPCR: *hisG*(+746→+853) fwd
JW8384	CAAGCGCTTTCAGTTTCTC	qPCR: *hisG*(+746→+853) rev
JW8405	AAGTCGAAGTTATCTACCGC	qPCR: *hisG*(+551→+665) fwd
JW8406	TGAATCACGCCCTGAATAC	qPCR: *hisG*(+551→+665) rev
JW8407	GTCTGTTAAATGGTTCTGTCG	qPCR: *hisG*(+446→+570) fwd
JW8408	GCGGTAGATAACTTCGACTT	qPCR: *hisG*(+446→+570) rev
JW8688	TTCATGCATTGCTAGCttaAATTAAATTACCCGCCTTGC	Cloning: *suhB*(←+57nt) rev
JW8817	AGAGACCGCAGCATCCGATGCTGAACATC	Cloning: *suhB*(A1C, T2A) mutation fwd
JW8818	GATGCTGCGGTCTCTCTCACTGGATG	Cloning: *suhB*(A1C, T2A) mutation rev

Supplementary References

1. Carlomagno, M. S. & Nappo, A. The antiterminator NusB enhances termination at a sub-optimal Rho site. *J Mol Biol* **309,** 19–28 (2001).
2. Carlomagno, M. S. & Nappo, A. NusA modulates intragenic termination by different pathways. *Gene* **308,** 115–28 (2003).
3. Berg, K. L., Squires, C. L. & Squires, C. Ribosomal RNA operon anti-termination. Function of leader and spacer region box B-box A sequences and their conservation in diverse micro-organisms. *J Mol Biol* **209,** 345–358 (1989).
4. Nodwell, J. R. & Greenblatt, J. Recognition of boxA antiterminator RNA by the E. coli antitermination factors NusB and ribosomal protein S10. *Cell* **72,** 261–268 (1993).
5. Blattner, F. R. *et al.* The complete genome sequence of Escherichia coli K-12. *Science* **277,** 1453–1462 (1997).
6. Stringer, A. M. *et al.* Genome-Scale Analyses of Escherichia coli and Salmonella enterica AraC Reveal Non-Canonical Targets and an Expanded Core Regulon. *J Bacteriol* **196,** 660–671 (2014).
7. Singh, N. *et al.* SuhB Associates with Nus Factors To Facilitate 30S Ribosome Biogenesis in Escherichia coli. *mBio* **7,** e00114-16 (2016).
8. Datsenko, K. A. & Wanner, B. L. One-step inactivation of chromosomal genes in Escherichia coli K-12 using PCR products. *Proc Natl Acad Sci USA* **97,** 6640–6645 (2000).
9. Guzman, L. M., Belin, D., Carson, M. J. & Beckwith, J. Tight regulation, modulation, and high-level expression by vectors containing the arabinose PBAD promoter. *J. Bacteriol.* **177,** 4121–4130 (1995).
