# Supplementary material for: Identification of regulatory targets for the bacterial Nus factor complex
Source: Nat Commun. 2017 Dec 11;8:2027. doi: 10.1038/s41467-017-02124-9 (PMC5725501; doi:10.1038/s41467-017-02124-9)
Supplement: Supplementary file 3 — Description of Additional Supplementary Files [file 41467_2017_2124_MOESM3_ESM.pdf]

Description of Additional Supplementary Files

File Name: Supplementary Data 1
Description: List of all annotated genes in γ-proteobacteria with a putative boxA sequence ≤50 bp upstream.
